# Waist circumference, among metabolic syndrome components, predicts degraded trabecular bone score: a retrospective study of a female population from the 2005-2008 NHANES cohorts

**DOI:** 10.3389/fendo.2024.1476751

**Published:** 2024-11-21

**Authors:** Maria Totaro, Ilaria Barchetta, Federica Sentinelli, Flavia Agata Cimini, Sara Palazzi, Francesco D’Alessandro, Luca Spagnolo, Sara Dule, Arcangelo Barbonetti, Maria Gisella Cavallo, Marco Giorgio Baroni

**Affiliations:** ^1^ Department of Clinical Medicine, Public Health, Life and Environmental Sciences (MeSVA), University of L’Aquila, L’Aquila, Italy; ^2^ Department of Experimental Medicine, Sapienza University, Rome, Italy; ^3^ Neuroendocrinology and Metabolic Diseases, IRCCS Neuromed, Pozzilli, Italy

**Keywords:** trabecular bone score, osteoporosis, fracture, metabolic syndrome, waist circumference

## Abstract

**Background:**

Osteoporosis and metabolic syndrome (MetS) are conditions associated with ageing and chronic inflammation; among MetS’ components, visceral obesity has been correlated to low bone mineral density in postmenopausal women. However, data on an increased fracture risk in MetS are still contrasting. The trabecular bone score (TBS) is an indicator of bone quality and a potential predictive factor for fractures. We aim to explore the relationship between MetS components and TBS.

**Methods:**

we analyzed data from 3962 women in the 2005-2006 and 2007-2008 NHANES cohorts, for whom a valid TBS value was available. All analyses were adjusted for the principal risk factors of altered bone metabolism.

**Results:**

An inverse significant association was observed between TBS and most of the MetS variables investigated, with the strongest correlation found with waist circumference (WC) (P <0.001). WC represented the major predictor of degraded TBS (P <0.001), in adjusted models considering age, 25(OH)Vitamin D, smoke and insulin resistance. Increased WC was significantly associated with the presence of bone fractures at the logistic regression analysis (P = 0.001) in all study participants and in the subgroup of women ≤50 years old after adjustment for potential confounders (P = 0.006).

**Conclusion:**

This study, using a large sample of women, found a negative association of MetS on bone health, mainly driven by visceral obesity.

## 1 Introduction

As life expectancy increases in industrialized countries, osteoporosis and fragility fractures are becoming major public health problems. According to current estimates, the world prevalence of osteoporosis is reported to be 18.3%, increasing to 23.1% in females (1), and The National Osteoporosis Foundation (NOF) estimates that more than 2 million osteoporosis-related fractures occur annually in the United States (US), most of them in women (70%) (2). Osteoporosis is conceptually defined as a progressive systemic skeletal disease characterized by low bone mass and microarchitectural deterioration of bone tissue, leading to increased bone fragility and increased risk of fractures (3). This definition includes the two pillars of bone resilience to fracture: bone density (quantitative property), and bone microarchitecture, or the material and organizational properties of bone (qualitative property) (4). The densitometric bone mass assessment of osteoporosis is based on bone mineral density (BMD) and measured using the densitometric assessment by dual-energy X-ray absorptiometry (DXA), the standard tool for the diagnosis of osteoporosis (5). However, BMD accounts only for 60%-70% of the variation in bone strength and the densitometric threshold (T-score of − 2.5 or less), is effective in identifying some, but not all individuals who go on to experience a fragility fracture (48; 6, 7). This indicates that factors other than bone mass influence bone strength and fracture risk, such as older age and personal history of osteoporotic fractures (49). Additionally, bone strength is also affected by the structural properties of bone (trabecular thickness, connectivity, separation and number, and cortical thickness and porosity), which are the second pillar of fracture resilience (4, 8). Bone microarchitecture can be investigated with a novel, non-invasive imaging technique, the trabecular bone score (TBS), a BMD-independent parameter evaluating pixel grey-level variations in lumbar spine DXA image. TBS provides a validated DXA-derived index of bone microarchitecture, correlates with the mechanical properties of bone, and according to an updated systematic review, is an independent predictor of incident fracture in 16 of 18 studies (4, 9). Osteoporosis is a condition associated with advancing age, as is another condition that characteristically occurs more frequently in adulthood, such as metabolic syndrome (MetS) (10). Studies have reported controversial results on the association of components of MetS, such as abdominal obesity, hypertriglyceridemia and low High-Density Lipoprotein-cholesterol (HDL-c), with not only low BMD in postmenopausal women (11–13), but also with bone fracture risk, so it is unclear whether MetS is a detrimental (14) or protective (50) risk factor for fractures. Also, the relationship between MetS and TBS was recently investigated in a small number of studies: some showed that the prevalence of degraded bone microarchitecture (TBS<1.23) (15) is significantly increased in subjects with MetS compared to subjects without (13) and that there is a significant negative association between TBS and more components of MetS, but only a few studies examined it with individual components, although in small populations (16, 17), so the relationship between MetS components and TBS remains unclear and warrants further studies. Since TBS is an indicator of bone quality and a potentially predictive factor for fractures, this study aimed to explore the relationship between MetS components and TBS in women, using data from the large female sample size of the 2005-2008 cohorts.

## 2 Methods

### 2.1 Study population

This study used data from two survey cohorts of the NHANES, which is a program of the National Center for Health Statistics (NCHS), as a part of the Centers for Disease Control and Prevention, to assess the health and nutritional status of a representative sample of the non-institutionalized, civilian population living in the US. Although a representative sample is currently collected each year in NHANES, data is released for 2-year periods to increase statistical reliability. Information regarding the participants, such as demographic information, medical examination results and questionnaires addressing medical and personal history was collected by trained examiners during household interviews. All medical and physical examinations were conducted at a Mobile Examination Centre (MEC) (18). Among all available NHANES cohorts, in this study we analyzed data sets from the 2005-2006 and the 2007-2008 cohorts, the only ones with TBS values and enough data on MetS components (19, 20). All procedures in NHANES 2005–2008 cohorts were approved by the NCHS Research Ethics Review Board and written informed consent was obtained from all subjects. The study population consisted of 5268 female adults from the 2005-2006 cohort and 5053 female adults from the 2007-2008 cohort. The inclusion criterion was the retrieval of a TBS value for the female adult population of the two NHANES cohorts: 6359 participants were therefore excluded because they lacked valid TBS data. The final study population consisted of 3962 female participants ages 20 years and older with valid data for TBS. Analyses were also conducted by dividing the participants into two subgroups based on the mean age of menopause in the US female population (≤ 50 years and > 50 years) (21).

### 2.2 Clinical and biochemical evaluations

Anthropometric data, such as age, waist circumference (WC), systolic blood pressure (SBP) and diastolic blood pressure (DBP), but also laboratory parameters, like total cholesterol, triglycerides, HDL-c, Low-Density Lipoprotein cholesterol (LDL-c), fasting blood glucose (FBG), fasting insulin, glycosylated hemoglobulin (HbA1c), used to asses glycemic control (22), aspartate aminotransferase (AST), alanine aminotransferase (ALT), 25(OH)Vitamin D, serum calcium, serum creatinine, serum phosphate, and medical history, including the presence of blood glucose alterations, evaluated as the presence of diabetes/use of hypoglycemic drugs, and presence/number of bone fractures, were retrieved from NHANES databases. Body mass index (BMI) was calculated as body weight (kilograms) divided by height (meters squared). WC was measured just above the uppermost lateral border of the right ilium while respondents were standing (23). Insulin Resistance (IR) was evaluated by the Homeostasis model assessment-IR (HOMA-IR) according to Matthews and colleagues (24). Specimen collection and processing procedures are provided in detail in the 2005-2006 and 2007-2008 NHANES Laboratory procedures Manual (19, 20).

### 2.3 DXA, TBS and mets assessment

Total spine DXA was measured from posterior-anterior (PA) spine scans obtained with Hologic QDR 4500A fan-beam densitometers (Hologic, Inc., Bedford, Massachusetts). Each of the raw DXA images of the spine was then uploaded to TBS iNsight^®^ version 2.1 software (Med-Imaps, Pessac, France). TBS is derived from the PA spine DXA image by evaluating pixel grey-level variations (25). Rigorous quality control (QC) programs were employed for DXA, which included the use of anthropomorphic phantoms and review of each QC and respondent scan at a central site (Department of Radiology of the University of California, San Francisco), using standard radiologic techniques and study-specific protocols developed for the NHANES (23). DXA scans of the spine were excluded if degenerative diseases, fusions or fractures, removable or non-removable implants and prostheses, excessive x-ray “noise” due to obesity, positioning problems or participant movement during the scan were noted on the image and if imaging procedure using contrast material in the previous seven days was self-reported by participants. BMD was expressed in g/cm^2^. Details of the DXA and TBS examination protocol have been described previously (19, 20, 23). We used the following cutoff points for TBS evaluation: TBS > 1.31 as normal, TBS between 1.23 and 1.31 for partially degraded microarchitecture and TBS < 1.23 indicating degraded microarchitecture (15). Based on the National Cholesterol Education Program’s Adult Treatment Panel III (NCEP: ATP III) study, MetS was defined as the presence of three or more of the following features: abdominal obesity as WC ≥102cm for men or ≥88 for women), serum triglyceride level ≥150mg/dl (1.7 mmol/L) or lipid-lowering medication; serum HDL-c <40mg/dl (1 mmol/L) for men or <50mg/dl (1.3 mmol/L) for women, high blood pressure as SBP ≥130mmHg or DBP ≥85 mmHg or on antihypertensive drugs; FBG ≥110 mg/dl (6.1 mmol/L) or on insulin or oral hypoglycemic agents (OHA) (51).

### 2.4 Statistical analysis

All statistical analysis was performed using the SPSS statistical package, version 27.0. Continuous data are shown as mean ± standard deviation (SD)/median and confidence interval (CI) of 95% and categorical variables as percentages. Pearson correlation coefficients were calculated to assess the strength and direction of the relationship between TBS and the variables examined in the present study, using not only those associated with MetS but also parameters associated with the control of bone metabolism, such as 25(OH) Vitamin D. The effect of MetS components and clinical and biochemical features on TBS, that showed significant association at the univariate/bivariate analyses, was examined using multivariate linear regression models and several extended models were established for covariate adjustment: model 1 was unadjusted; model 2 was adjusted for age; model 3 was adjusted for age and 25(OH)Vitamin D; model 4 was adjusted for age, 25(OH)Vitamin D and smoke; model 5 was adjusted for age, 25(OH)Vitamin D, smoke and HOMA-IR. Multivariate linear regression analyses were performed in the entire population and, also, dividing women into two groups by age, ≤50 years and >50 years. In addition, univariate and multivariate logistic regression analyses for a history of fractures (≥1) were performed in all the study participants and in the women ≤50 years and >50 years. P values <0.05 were considered statistically significant with a CI of 95%.

## 3 Results

### 3.1 Characteristics of the study population

A total of 3962 female participants (mean age of 49.9 years) were enrolled: 29.3% of the women were overweight with BMI between 25 and 29.9kg/m^2^), 38.2% were obese (BMI ≥30kg/m^2^), 21.7% had impaired fasting glucose (IFG) with FBG≥100mg/dl and mean HOMA-IR value was indicative for insulin-resistance (HOMA 3.2 ± 1.0). 12.6% of the study population reported having diabetes or taking hypoglycemic drugs during household interviews. TBS values indicated a partially degraded bone microarchitecture for 14.9% and a degraded bone microarchitecture for 19.4% of the study population. The main clinical and biochemical features of the study population are summarized in **Table 1**.

**Table 1:** Clinical and biochemical features of the study population (n=3962).

Clinical and biochemical features	
Age (years)	49.9 ± 17.4
BMI (Kg/m^2^)	28.8 ± 6.6
BMI<25kg/m^2^	1289 (32.5)
BMI=25-29.9kg/m^2^	1160 (29.3)
BMI ≥30kg/m^2^	1513 (38.2)
WC (cm)	95.4 ± 15.1
Smoke	1759 (19.2)
TBS	1.4 (1.3, 1.5)
TBS>1.31 (normal)	2601 (65.7)
TBS=1.23-1.31 (partially degraded)	592 (14.9)
TBS ≤ 1.23 (degraded)	769 (19.4)
BMD (g/cm^2^)	1.0 (0.9, 1.1)
SBP (mmHg)	121.4 ± 19.7
DBP (mmHg)	69.1 ± 11.5
FBG (mg/dL)	106.7 ± 36.9
FBG≥100mg/dl (IFG)	859 (21.7)
HbA1c (%)	5.6 ± 1.0
Insulin (uU/mL)	12.1 ± 10.8
HOMA-IR	3.2 ± 1.0
Total cholesterol (mg/dL)	200.4 ± 41.3
HDL-c (mg/dL)	57.7 ± 16.4
LDL-c (mg/dL)	114.9 ± 36.1
Triglycerides (mg/dL)	143.0 ± 110.7
AST (IU/L)	24.6 ± 19.9
ALT (IU/L)	22.4 ± 20.8
Serum Creatinine (mg/dL)	0.8 ± 0.3
eGFR (EPI-CKD) (ml/min/1.73 m^2^)	93.1 ± 22.8
25(OH)Vitamin D (nmol/L)	59.6 ± 24.9
Serum calcium (mg/dL)	9.4 ± 0.4
Serum phosphate (mg/mL)	3.9 ± 0.5

Data are shown as mean values ± standard deviation (SD) or median (25, 75 percentile) or number (percentages).

AST, Aspartate Aminotransferase; ALT, alanine aminotransferase; BMD, Bone Mineral Density; BMI, Body Mass Index; DBP, Diastolic Blood Pressure; eGFR, Estimated Glomerular Filtration Rate; FBG, Fasting Blood Glucose; HbA1c, Glycosylated Hemoglobin; HDL, High-Density Lipoprotein; HOMA-IR, Homeostasis Model Assessment-Insulin Resistance; IFG, Impaired Fasting Glucose; LDL, Low-Density Lipoprotein; SBP, Systolic Blood Pressure; TBS, Trabecular Bone Score; WC, Waist circumference.

### 3.2 Clinical and metabolic correlates of degraded TBS in study population

When exploring the relationship between bone architecture and clinical/metabolic parameters, an inverse significant association was observed between TBS and most of the variables investigated, with a stronger negative correlation with WC (r = -0.579; P <0.001). TBS, also, showed a strong inverse association with age (r = -0.520; P <0.001), BMI (r = -0.495; P <0.001), total spine BMD (r =-0.406; P<0.001), HbA1c (r = -0.392; P <0.001), SBP (r = -0.358; P <0.001), FBG (r = -0.297; P <0.001), triglycerides (r = -0.273; P <0.001), total cholesterol (r = -0.151; P <0.001), LDL-c (r = -0.108; P <0.001) and insulin resistance degree, as estimated by HOMA-IR (r = -0.28, P <0.001). On the other hand, TBS positively correlated with HDL-c (r = 0.125; P <0.001), serum 25(OH)Vitamin D levels and Estimated Glomerular Filtration Rate (eGFR) (r = 0.38, P <0.001). At the multivariate regression analysis, greater WC resulted in the best predictor for degraded TBS (standardized β coefficients = -0.58, P <0.001) in adjusted models for age, 25(OH)Vitamin D, smoking status, eGFR and HOMA-IR (**Table 2**). The same results were obtained when dividing the study population according to the mean age of menopause in the US female population: WC resulted in the best predictor for degraded TBS in women ≤50 years (standardized β coefficients = -0.64, P <0.001) and in women >50 years (standardized β coefficients = -0.53, P <0.001), in unadjusted and adjusted models (**Tables 3**, **4**). Since a possible effect on TBS has been hypothesized for soft tissue, which absorbs more X-rays during DXA acquisition and might result in lower “raw” TBS values (26), we tested WC in subgroups of BMI. WC remained negatively associated with TBS also when dividing the study population according to BMI in multivariate adjusted models for age, 25(OH)Vitamin D, smoking status and eGFR: in obese women with BMI ≥30 kg/m^2^ (standardized β coefficients = -0.530, P <0.0001), in overweight women with BMI between 25 and 29.9 kg/m^2^ (standardized β coefficients = -0.197, P <0.0001) and even in women with BMI <25 kg/m^2^ (standardized β coefficients = -0.047, P <0.035).

**Table 2:** Multivariate linear regression models for TBS value in the entire study population.

Model	Variables	Non standardized coefficient		Standardized β coefficients	*P* value
		β	SE		
1	Constant	1.911	0.019	–	<0.001
	WC	-0.006	0.000	-0.58	<0.001
2	Constant	2.043	0.016	–	<0.001
	WC	-0.005	0.000	-0.51	<0.001
	Age	-0.004	0.000	-0.46	<0.001
3	Constant	1.984	0.019	–	<0.001
	WC	-0.005	0.000	-0.48	<0.001
	Age	-0.004	0.000	-0.47	<0.001
	25(OH)Vitamin D	0.001	0.000	0.10	<0.001
4	Constant	1.990	0.019	–	<0.001
	WC	-0.005	0.000	-0.47	<0.001
	Age	-0.004	0.000	-0.47	<0.001
	25(OH)Vitamin D	0.001	0.000	0.10	<0.001
	Smoke	-0.020	0.006	-0.05	<0.001
5	Constant	1.980	0.020	–	<0.001
	WC	-0.005	0.000	-0.47	<0.001
	Age	-0.004	0.000	-0.47	<0.001
	25(OH)Vitamin D	0.001	0.000	0.10	<0.001
	Smoke	-0.020	0.006	-0.05	<0.001
	HOMA-IR	-0.001	0.001	-0.04	0.021

TBS is the dependent variable. Model 1 R: 0.580, R^2^: 0.337; Model 2 R: 0.738, R^2^: 0.545; Model 3 R: 0.744, R^2^: 0.554; Model 4 R: 0.746, R^2^: 0.557; Model 5 R: 0.747, R^2^: 0.558. eGFR is not present in table because of collinearity with age.

HOMA-IR, Homeostasis model assessment-Insulin resistance; SE, standard error; WC, Waist circumference.

**Table 3:** Multivariate linear regression analyses for TBS value in women ≤50 years.

Model	Variables	Non standardized coefficient		Standardized β coefficients	*P* value
		β	SE		
1	Constant	1.917	0.020	–	<0.001
	WC	-0.005	0.000	-0.64	<0.001
2	Constant	1.995	0.022	–	<0.001
	WC	-0.005	0.000	-0.62	<0.001
	Age	-0.003	0.000	-0.19	<0.001
3	Constant	1.955	0.025	–	<0.001
	WC	-0.005	0.000	-0.59	<0.001
	Age	-0.003	0.000	-0.20	<0.001
	25(OH)Vitamin D	0.000	0.000	0.08	<0.001
4	Constant	1.952	0.025	–	<0.001
	WC	-0.005	0.000	-0.59	<0.001
	Age	-0.003	0.000	-0.19	<0.001
	25(OH)Vitamin D	0.000	0.000	0.09	<0.001
	Smoke	-0.018	0.007	-0.060	0.017

TBS is the dependent variable. Model 1 R: 0.640, R^2^: 0.409; Model 2 R: 0.667, R^2^: 0.445; Model 3 R: 0.672, R^2^: 0.452; Model 4 R: 0.675 R^2^: 0.455.

HOMA-IR, Homeostasis model assessment-Insulin resistance; SE, standard error; WC, Waist circumference.

**Table 4:** Multivariate linear regression analyses for TBS value in women >50 years.

Model	Variables	Non standardized coefficient		Standardized β coefficients	*P* value
		β	SE		
1	Constant	1.770	0.027	–	<0.001
	WC	-0.005	0.000	-0.53	<0.001
2	Constant	2.000	0.038	–	<0.001
	WC	-0.005	0.000	-0.55	<0.001
	Age	-0.003	0.000	-0.23	<0.001
3	Constant	1.913	0.041	–	<0.001
	WC	-0.005	0.000	-0.51	<0.001
	Age	-0.003	0.000	-0.24	<0.001
	25(OH)Vitamin D	0.001	0.000	0.16	<0.001
4	Constant	1.938	0.041	–	<0.001
	WC	-0.005	0.000	-0.52	<0.001
	Age	-0.003	0.000	-0.25	<0.001
	25(OH)Vitamin D	0.001	0.000	0.15	<0.001
	Smoke	-0.030	0.011	-0.08	0.005
5	Constant	2.009	0.052	–	<0.001
	WC	-0.005	0.000	-0.52	<0.001
	Age	-0.004	0.000	-0.28	<0.001
	25(OH)Vitamin D	0.001	0.000	0.15	<0.001
	Smoke	-0.030	0.010	-0.77	0.006
	eGFR	0.000	0.000	-0.71	0.025
6	Constant	1.998	0.052	–	<0.001
	WC	-0.005	0.000	-0.50	<0.001
	Age	-0.004	0.000	-0.28	<0.001
	25(OH)Vitamin D	0.001	0.000	0.15	<0.001
	Smoke	-0.030	0.010	-0.77	0.006
	eGFR	0.000	0.000	-0.73	0.020
	HOMA-IR	-0.002	0.001	-0.64	0.028

TBS is the dependent variable. Model 1 R: 0.534, R^2^: 0.285; Model 2 R: 0.583, R^2^: 0.340; Model 3 R: 0.602, R^2^: 0.363; Model 4 R: 0.607, R^2^: 0.369; Model 5 R: 0.610, R^2^: 0.372; Model 6 R: 0.613, R^2^: 0.376.

HOMA-IR, Homeostasis model assessment-Insulin resistance; SE, standard error; WC, Waist circumference.

### 3.3 Logistic regression analysis for history of fractures in study population

As TBS is a potential predictor for bone fractures, we performed univariate and multivariate logistic regression analysis for a history of fractures (≥1): impaired TBS was significantly associated with fractures (β coefficient = -2.207; OR(95% CI) = 0.11(0.07, 0.19); P <0.001) and this association is independent of total spine BMD (β co-efficient = -1.834; OR(95% CI) = 0.16(0.09, 0.28); P <0.001). Moreover, since TBS is a better predictor for bone fractures than total spine BMD and WC is the only MetS component strongly associated with TBS and the best predictor for degraded TBS, we also performed multivariate logistic regression analysis for WC and history of fractures. WC was significantly associated with bone fractures (β coefficient = 0.011; OR(95% CI) = 1.01(1.00, 1.0); P = 0.001) in all study participants (**Table 5**) and, also, unexpectedly, in women group ≤50 years at univariate analysis (β coefficient = 0.009; OR(95% CI) = 1.01 (1.00, 1.02); P = 0.007). Therefore, the association between WC and fractures is confirmed to be significant even after adjustment for potential confounders, such as TBS, total spine BMD, age, HbA1c, eGFR and smoke at multivariate logistic regression analysis (β coefficient = 0.013; OR (95% CI) = 1.01(1.00, 1.02); P = 0.006), as shown in **Table 6**. In women >50 years WC did not predict bone fractures at univariate (β coefficient = -0.002; OR(95% CI) = 0.998(0.99, 1.01); P = 0.53) and multivariate logistic regression analysis (β coefficient = 0.007; OR(95% CI) = 1.01(0.99, 1.02); P = 0.19) and the effect of age (β coefficient = 0.022; OR(95% CI) = 1.02(1.01, 1.04); P = 0.003), HbA1c (β coefficient = -0.200; OR(95% CI) = 0.82(0.72, 0.93); P = 0.001) and smoke (β coefficient = 0.466; OR(95% CI) = 1.59(1.18, 2.16); P = 0.003) was, instead, predominant.

**Table 5:** Multivariate logistic regression analysis for WC and history of fractures (≥1) in all the study participants.

Multivariate	Fractures			
	β	SE	OR (95% CI)	*P* value
WC	0.011	0.003	1.01 (1.00, 1.02)	0.001
TBS	-0.022	0.126	0.98 (0.76, 1.25)	0.86
Total spine BMD	-0.756	0.313	0.47 (0.25, 0.87)	0.02
Age	0.028	0.004	1.03 (1.02, 1.04)	<0.001
HbA1c	-0.195	0.054	0.82 (0.74, 0.92)	<0.001
eGFR	-0.003	0.003	1.00 (0.99, 1.002)	0.26
Smoke	0.753	0.097	2.12 (1.76, 2.57)	<0.001

BMD, Bone Mineral Density; BMI, Body Mass Index; eGFR, Estimated Glomerular Filtration Rate; HbA1c, Glycosylated Hemoglobin; OR, Odds Ratio; SE, Standard Error; TBS, Trabecular Bone Score; WC, Waist circumference.

**Table 6:** Multivariate logistic regression analysis for WC and history of fractures (≥1) in women ≤50 years.

Multivariate	Fractures			
	β	SE	OR (95% CI)	*P* value
WC	0.013	0.005	1.01 (1.00, 1.02)	0.006
TBS	-0.157	0.256	0.86 (0.52, 1.41)	0.54
Total spine BMD	-1.486	0.501	0.23 (0.09, 0.61)	0.003
Age	0.035	0.008	1.04 (1.02, 1.05)	<0.001
HbA1c	-0.194	0.108	0.82 (0.67, 1.02)	0.07
eGFR	-0.007	0.004	0.99 (0.98, 1.00)	0.07
Smoke	0.914	0.126	2.50 (1.95, 3.19)	<0.001

BMD, Bone Mineral Density; BMI, Body Mass Index; eGFR, Estimated Glomerular Filtration Rate; HbA1c, Glycosylated Hemoglobin; OR, Odds Ratio; SE, Standard Error; TBS, Trabecular Bone Score; WC, Waist circumference.

## 4 Discussion

The main result of this study is that increased waist circumference predicts impaired TBS and, also, greater fracture risk in women under 50 years old. We also found that degraded TBS is detectable in the presence of other individual components of the metabolic syndrome (FBG, SBP, triglycerides) and its. TBS positively correlates, instead, only with HDL-c, as already reported (27), but with a weaker association. The impact of MetS on bone health is still controversial, particularly in clinical studies assessing BMD, in which the protection provided by increased body weight is balanced by the damage due to the inflammatory state, hyperglycemia and IR (28). In Romagnoli and colleagues’ study, subjects with MetS showed, on average, a lower value of TBS (17). Also, the study of Shih et al., analyzing data from both men and women of the 2005-2006 NHANES cohort, demonstrated a negative relationship between TBS and an increased number of MetS components (three or more in men and four or more in women) in an adjusted model of both sexes. Also, similar to our results, the authors showed in both sexes an association between fasting glucose level and low TBS, and with smaller beta coefficients with abdominal obesity, high SBP, and high serum triglycerides (16). In our study, WC results as the best predictor for degraded TBS in women at multivariate linear regression analyses, even in adjusted models. Also in obese men, consistent with our results, Romagnoli et al. revealed that WC had a more pronounced effect on TBS than BMI and that WC, instead of BMI, should be probably considered in men, when assessing TBS performance (17). However, TBS was not significantly associated with MetS in either sex in Bagherzadeh and colleagues’ work, after including BMI in the adjusted models (13). As some studies hypothesized, BMI or body weight could be the main factors that determined the effect of MetS on BMD and, after adjustment for it, the positive effect of MetS tended to disappear (29). Traditionally, obesity was regarded as a protective factor against osteoporosis and a positive association between BMI and BMD was reported (30). Furthermore, BMI may not be related to adipose tissue distribution, which is known to affect bone health. Indeed, several studies revealed that visceral obesity, mirrored by WC, may be associated with impaired bone microarchitecture, and affect skeletal health more than general obesity, reflected by BMI (31–34). In the study of Jose et al., the bone microarchitecture, evaluated with TBS, was found to be significantly lower in postmenopausal women with morbid obesity (BMI ≥ 35 kg/m^2^) as compared to obese (BMI 25-35 kg/m^2^) and non-obese (BMI ≤ 25kg/m^2^) age-matched women (34). Furthermore, Gilsanz and colleagues found that even in healthy young women with weights between the 3rd and 97th percentiles, visceral abdominal fat is negatively correlated with the amount and strength of bone in the appendicular skeleton, supporting the hypothesis that visceral fat serves as a pathogenic fat depot for bone health (33). Finally, a recent biomechanical study showed that increased WC at the same body weight leads to increased pressure on the spine with a higher risk for low-trauma and compression fracture (52). Several mechanisms have been hypothesized to explain the positive relationship between adipose tissue and bone metabolism, from mechanical loading (30) to dysregulation of several adipokines, including leptin and adiponectin and higher insulin and 17β-estradiol levels, as the main mechanisms by which abdominal obesity contributes to higher BMD in obese individuals (35). On the other hand, visceral adipose tissue produces a higher number of inflammatory cytokines than subcutaneous fat tissue (36), such as interleukin-6 (IL-6), which promotes osteoclast differentiation/activation and stimulates the production of Tumor necrosis factor-alpha (TNF-alpha), leading to increased osteoclastogenesis and bone resorption (37). Additionally, central adiposity is associated with dysregulation of the growth hormone (GH)/insulin-like growth factor (IGF)-1 axis with lower serum IGF-1 level, which may adversely affect bone formation and cause poor bone quality (31). Moreover, visceral fat produces the 11β-hydroxysteroid dehydrogenase type 1(11β-HSD1) enzyme that converts inactive cortisone to active cortisol, negatively impacting BMD and increasing the risk of bone fractures (38, 39). Finally, people with abdominal obesity generally exercise less, which has negative effects on bone health (40). All these mechanisms, driven by central obesity, may have detrimental effects on bone remodeling and reduce bone quality. Finally, this reported negative correlations between WC and TBS could be related to the effects of soft tissue artifact, rather than TBS reflecting an alteration in “bone health”: increased amounts of abdominal soft tissue, such as in individuals with central adiposity and elevated BMI, absorb more X-rays during DXA acquisition and will result in lower “raw” TBS values (26). To compensate for this effect that is in part BMI-based, adjustments have been incorporated in the TBS algorithm (41) and TBS measurement has been optimized for individuals with a BMI in the range of 15–37 kg/m^2^ (26). To ensure that this “artifact” was not at play, we performed regression analysis dividing the study population according to BMI subgroups in adjusted models for age, 25(OH)Vitamin D, smoking status and WC resulted negatively correlated with TBS, even in women with BMI <25 kg/m^2^ (standardized β coefficients = -0.047, P <0.035), in which the effect of soft tissue artifact could be negligible, given the lower presence and thickness of adipose tissue. Moreover, in our study, degraded TBS was significantly associated with bone fractures and this association is independent of total spine BMD. This finding is consistent with the scientific literature in post-menopausal women as well as in men over the age of 50 years (15, 42–44) and confirmed in a 2023 systematic review by the Working group of the European Society for Clinical and Economic Aspects of Osteoporosis, Osteoarthritis and Musculoskeletal Diseases (ESCEO) and the International Osteoporosis Foundation (IOF) (4). Furthermore, our results demonstrate that WC is significantly associated with bone fractures in all study participants and, also, unexpectedly, in the women group ≤50 years, even after adjustment for potential confounders, such as TBS and BMD, thus reinforcing the hypothesis that the possible negative effect of MetS on bone quality is mainly driven by WC. This hypothesis is confirmed by studies from other authors (12, 13, 17, 29) and is consistent with the results of a very recent meta-analysis, which found that abdominal obesity was associated with an increased risk of vertebral fracture and that each 10 cm increase in WC was associated with a 3% higher risk of vertebral fracture (45). This significant association found in our study between WC and bone fracture in US women, particularly in women ≤50 years, has important clinical relevance, as it draws attention to the benefit of a healthy lifestyle and, consequently, of weight reduction in obese people, as a factor that may have a positive impact on bone health and on fracture risk management. In women >50 years, on the other hand, WC does not predict bone fractures at univariate and multivariate logistic regression analysis and the effect of age and glycaemic control (FBG and HbA1c) is predominant. A negative correlation between FBG levels and TBS has been revealed in previous studies (17, 46) and the possible mechanisms may be related to the dysregulation of IGF-1 in individuals with impaired glucose tolerance (IGT), as already mentioned, and to the accumulation of advanced glycation end products in the organic bone matrix and to low bone turnover in patients with diabetes (46). However, Holloway and colleagues reported that the correlation between TBS and blood glucose was only present in diabetic individuals and there was no difference in TBS between those with normoglycemia and those with IFG (47), so further studies are needed to determine the impact of IFG and diabetes on TBS. The finding that WC does not predict bone fractures in women >50 years, can be explained, hypothetically, as the result of the effect of increased WC over many years, which is manifested by the development, later in life, of glycemic alterations and diabetes, which are, indeed, related to central obesity and, therefore, to increased WC.

This study has some limitations. Firstly, the cross-sectional design of the study allows investigating for association and not for causality. Moreover, due to the retrospective design of the study, not all confounding factors have been considered, due to the lack or a small number of these parameters (levels of bone turnover markers, thyroid and sex hormones) Finally, the effects of soft tissue artifacts cannot be ruled out, although we performed linear regression analysis adjusted for BMI subgroups, showing that WC association with TBS was present across all BMIs, from lean to overtly obese.

## 5 Conclusion

In conclusion, this study, strengthened by including a large representative sample of the NHANES cohort women, emphasizes that WC, among individual components of MetS, shows the strongest association with degraded TBS, and that WC is significantly associated with bone fractures, thus reinforcing the assumption that the possible negative association of MetS on TBS is mainly driven by WC, especially in younger women. This significant association between WC and bone fracture in US women ≤50 years has clinical relevance, as it draws attention to the benefit of a healthy lifestyle and, consequently, of weight control, as a factor that may have a positive impact on bone health and fracture risk management even at younger ages. Further studies are needed to determine the net impact of measures of visceral adipose tissue on bone health and fracture outcome and to investigate other possible determinants of TBS.

## Data Availability

The original contributions presented in the study are included in the article/supplementary material. Further inquiries can be directed to the corresponding author/s.
